# A Systematic comparison of *in vitro* cell uptake and *in vivo* biodistribution for three classes of gold nanoparticles with saturated PEG coatings

**DOI:** 10.1371/journal.pone.0234916

**Published:** 2020-07-02

**Authors:** Yijia Zhang, Alice T. Liu, Yvonne R. Cornejo, Desiree Van Haute, Jacob M. Berlin

**Affiliations:** Department of Molecular Medicine, Irell and Manella Graduate School of Biological Sciences, City of Hope National Medical Center, Beckman Research Institute, Duarte, California, United States of America; Consiglio Nazionale delle Ricerche, ITALY

## Abstract

A great deal of attention has been focused on nanoparticles for cancer therapy, with the promise of tumor-selective delivery. However, despite intense work in the field over many years, the biggest obstacle to this vision remains extremely low delivery efficiency of nanoparticles into tumors. Due to the cost, time, and impact on the animals for *in vivo* studies, the nanoparticle field predominantly uses cellular uptake assays as a proxy to predict *in vivo* outcomes. Extensive research has focused on decreasing macrophage uptake *in vitro* as a proxy to delay nanoparticle accumulation in the mononuclear phagocytic system (MPS), mainly the liver and spleen, and thereby increase tumor accumulation. We have recently reported novel synthetic methods employing small molecule crosslinkers for the controlled assembly of small nanoparticles into larger aggregates and found that these nanoaggregates had remarkably high surface coverage and low cell uptake, even in macrophages. We further found that this extremely low cellular uptake could be recapitulated on solid gold nanoparticles by densely coating their surface with small molecules. Here we report our studies on the biodistribution and clearance of these materials in comparison to more conventional PEGylated gold nanoparticles. It was expected that the remarkably low macrophage uptake *in vitro* would translate to extended blood circulation time *in vivo*, but instead we found no correlation between either surface coverage or *in vitro* macrophage cell uptake and *in vivo* blood circulation. Gold nanoaggregates accumulate more rapidly and to a higher level in the liver compared to control gold nanoparticles. The lack of correlation between *in vitro* macrophage uptake and *in vivo* blood circulation suggests that the field must find other *in vitro* assays to use as a primary proxy for *in vivo* outcomes or use direct *in vivo* experimentation as a primary assay.

## Introduction

Nanoparticles have many applications in biomedicine such as biosensors, imaging contrast agents, or drug delivery systems [1]. However, those applications often require intravenous administration. Big obstacles for systemic application of nanoparticles are the low efficiency of targeting to disease locations[2] and the high accumulation and persistence in mononuclear phagocytic system (MPS), especially the liver, which potentially can cause toxicities to those healthy organs[3, 4]. It is commonly accepted that the high accumulation in MPS is driven by cellular uptake by phagocytic cells such as Kupffer cells in the liver[3, 4]. Thus, *in vitro* cellular uptake by macrophages has been widely used to predict MPS accumulation *in vivo*. Lower cellular uptake is generally regarded as predictive for reduced MPS accumulation. One further assumption in the field is that when lowering the clearance of nanoparticles by MPS, the delivery to disease tissues such as tumor will be increased.

Over the past several years, we have reported novel synthetic methods employing small molecule crosslinkers for the controlled assembly of small nanoparticles into larger aggregates and found that these nanoaggregates had remarkably high surface coverage and low cell uptake, even in macrophages. The aggregates were synthesized by crosslinking 5, 10, 15 or 20 nm AuNPs with either (pentaerythritol tetrakis(3-mercaptopropionate) (PTMP) or trimethylol tris(3-mercaptoproprionate) (TTMP) followed by reaction termination with PEG_2K_-maleimide (PEG-mal)[5, 6]. Intriguingly, cell uptake studies in multiple cell lines demonstrated that aggregates 60 nm in hydrodynamic diameter that were assembled from 5 nm starting particles had approximately 10-fold lower cell uptake compared to solid PEGylated AuNPs of the same size (**S1A Fig in S1 File**)[7]. It was found that the nanoaggregates had much greater surface coverage than the solid PEGylated AuNPs of the same size, so we hypothesized that this was responsible for the reduced *in vitro* cell uptake. Indeed, when the solid AuNPs were densely coated with PTMP and then PEGylated, their *in vitro* cell uptake was inversely correlated with degree of surface coverage [7]. In particular, AuNPs with high density of PTMP on surface, denoted as high surface coverage (HSC) AuNPs, had similarly low cellular uptake as aggregates. In this report, we detail our studies evaluating if this markedly reduced *in vitro* uptake for nanoaggregates and HSC AuNPs would translate to reduced MPS accumulation *in vivo* and hence, enhanced blood circulation.

In our pilot study of investigating *in vitro/in vivo* correlation, aggregates with hydrodynamic diameter of 60 nm were synthesized with a PEG_2K_ coating following our published protocols[5, 6]. HSC AuNPs and PEG AuNPs made of 50 nm AuNPs were included as control groups. In contrast to the marked difference between these groups for *in vitro* cell uptake, all of these materials were almost completely cleared from the blood at 1 h and the majority accumulated in the liver (**S1B Fig in S1 File**). Surprised by this result, we used 50 nm solid AuNPs as model system and systemically optimized PEG coating to ensure a saturated PEG coating. The parameters we evaluated were PEG length, density, and backfilling on nanoparticle to elongate blood circulation and decrease liver accumulation. We found that increasing PEG length (MW 2k to 20k) significantly improved nanoparticle biodistribution; all types of particles were fully coated and tuning PEG density and backfilling in the over-saturating range did not affect biodistribution. However, even with optimized PEGylation, relative to PEGylated solid AuNPs with very similar physicochemical properties, the aggregates showed reduced blood circulation and increased liver accumulation–though the aggregates did show modestly improved clearance from the liver. Altogether, our biodistribution results suggested *in vitro* cellular uptake did not translate to *in vivo* blood half-life and liver accumulation, which argues for the necessity of new *in vitro* predictive models or exclusively *in vivo* assays for biodistribution study of nanomaterials.

## Materials and methods

### Materials

Citrate-stabilized gold colloid suspensions (5 and 50 nm) were purchased from Ted Pella. Pentaerythritol tetrakis(3-mercaptopropionate) (PTMP) and trimethylol tris(3-mercaptoproprionate) (TTMP) were purchased from Sigma-Aldrich. Methoxy-PEG-thiol (PEG-SH) of varying molecular weights (MW 1k, 2k, 5k, 10k, and 20k) and methoxy-PEG-maleimide (PEG_20k_-mal) (MW 20k) were purchased from Rapp Polymere. ICP-MS grade nitric acid (70%) and hydrochloric acid (35%) were purchased from BD Aristar. ICP-MS gold and magnesium calibration solution (100 μg mL-1) was purchased from Spex CertiPrep. All other chemical compounds were purchased from Sigma-Aldrich.

### Synthesis of 50 nm PEGylated AuNPs

For PEG length experiment, a solution of PEG-SH in diH_2_O (20 mg mL^-1^) was prepared for each of the varying lengths (2k, 5k, 10k, 20k). The amount of PEG-SH added to the reaction solution was calculated based on an equivalent number of thiols as used for synthesizing PTMP aggregates (denoted as 1x concentration) (**Table 1**).

**Table 1:** Reagent calculations for PEGylation of 50 nm AuNPs with varying length PEG-SH at 1x concentration.

PEG MW (g mol^-1^)	eq. PTMP (mM)	mol PTMP (x10^-8^)	mol thiols (x10^-7^)	PEG-SH added (mg)	Volume PEG-SH (20 mg mL^-1^ H_2_O) (μL)	PEG per nm^2^
2000	0.12	9.47	3.79	0.76	37.9	1292.5
5000	0.12	9.47	3.79	1.89	94.7	1292.5
10000	0.12	9.47	3.79	3.79	189.4	1292.5
20000	0.12	9.47	3.79	7.58	378.8	1292.5

For PEG density experiment, 0.005x, 0.1x, 1x, and 10x concentrations of PEG_20k_-SH were also tested (based on the same denotation as above); see **Table 2** for example calculations for PEG_20k_-SH. 0.005x was chosen as the lowest concentration to match the PEG per nm^2^ used in previously reported literature[8]. For each reaction, 500 μL of stock 50 nm gold colloid (5.4 x 10^10^ particles mL^-1^) was added dropwise to the PEG-SH solution (20 mg mL^-1^ in diH_2_O) in a 2 mL Eppendorf tube. The reaction solution was shaken at room temperature on a tabletop shaker (GeneMate Rocker, variable speed, BioExpress) at top speed for 2 h.

**Table 2:** Reagent calculations for PEGylation of 50 nm AuNPs with PEG_20k_-SH at varying concentrations.

PEG MW (g mol^-1^)	PEG Concentration	mol thiols (x10^-7^)	PEG-SH added (mg)	Volume PEG-SH (20 mg mL^-1^ H_2_O) (μL)	PEG per nm^2^
20000	0.005x	0.02	0.04	1.9	6.5
20000	0.1x	0.38	0.76	37.9	129.3
20000	1x	3.79	7.58	378.8	1292.5
20000	10x	37.9	75.76	757.6 (100 mg mL^-1^)	12924.8

For PEG back-filling experiment, solutions of PEG_1k_-SH in diH_2_O were prepared at either 0.2 mg mL^-1^ or 20 mg mL^-1^ concentrations and rapidly added to nanoparticle solutions immediately after the particles finished shaking. Various concentrations of PEG_1k_-SH were tested, where 1x concentration is the same number of PEG per nm^2^ as denoted in Table 2; see **Table 3**for reagent calculations. After addition of PEG_1k_-SH, reaction solutions were placed back onto the tabletop shaker and shaken at room temperature at top speed for an additional 2 h. Particles were filtered using a 25 mm 0.2 μm polycarbonate track etch membrane in a Swinnex housing. The filtrate was washed three times by centrifugation (10 000 x g for 10 min) and resuspended in diH_2_O.

**Table 3:** Reagent calculations for back-filling 50 nm AuNPs with PEG_1k_-SH at varying concentrations.

PEG MW (g mol^-1^)	PEG Concentration	mol thiols (x10^-7^)	PEG-SH added (mg)	Volume PEG-SH in H_2_O (μL)	PEG per nm^2^
1000	0.005x	0.019	0.0019	9.47 (0.2 mg mL^-1^)	6.46
1000	1x	3.79	0.38	18.9 (20 mg mL^-1^)	1292.5
1000	10x	37.9	3.79	189.4 (20 mg mL^-1^)	12924.8

### Synthesis of AuNP aggregates

#### Aggregation using PTMP and TTMP crosslinkers

Aggregates were synthesized out of 5 nm AuNPs using either PTMP or TTMP crosslinkers. A standard reaction used 500 μL of stock 5 nm gold colloid in water (5 x 10^13^ particles mL^-1^) in a final volume of 789.2 μL. The final concentrations of PTMP and TTMP used were 0.12 mM and 0.20 mM, respectively. First, each crosslinker was dissolved in ethanol at a concentration of 4 mg mL^-1^. The appropriate volume of crosslinker solution was added to a 2 mL Eppendorf tube containing 173.2 μL diH_2_O (**Table 4**). Additional ethanol was added to keep the total volume of ethanol constant (116 μL total). 500 μL of stock 5 nm gold colloid was then added dropwise into the crosslinker-ethanol-water solution. The reaction solution was shaken at room temperature on a tabletop shaker (GeneMate Rocker, variable speed, BioExpress) at top speed for 2 h. After shaking, the solution was placed on the benchtop at room temperature for 24 h.

**Table 4:** Reagent calculations for synthesizing 50 nm aggregates using PTMP and TTMP crosslinkers and 5 nm AuNPs.

Linker	Final Aggregate size	Final [Linker] (mM)	Volume AuNPs (μL)	[Linker] in ethanol (mg mL^-1^)	Volume linker solution (μL)	Additional ethanol (μL)	Volume H_2_O (μL)
PTMP	60 nm	0.12	500	4	11.6	104.4	173.2
TTMP	60 nm	0.20	500	4	15.7	100.3	173.2

#### PEG-maleimide capping

After resting on the benchtop, the reaction was terminated by addition of PEG_20k_-mal. See **Table 5**below for an example calculation for aggregates. (Note: 1x concentration would denote the concentration of maleimides needed to cap every thiol of input in the reaction solution, which we set at 1.1 maleimides for every thiol.)

**Table 5:** Reagent calculations for varying the concentration of PEG_20k_-maleimide to cap aggregates synthesized at the standard 500 μL AuNP scale.

Linker	Final [Linker] (mM)	mol Linker (x10^-8^)	mol Thiols (x10^-7^)	PEG_20k_-mal concentration	mol PEG_20k_-mal needed (x10^-7^)	PEG_20k_-mal added (mg)	Volume PEG-mal (100 mg mL^-1^ H_2_O) (μL)
PTMP	0.12	9.47	3.79	0.1x	4.17	0.83	8.3
PTMP	0.12	9.47	3.79	1x	0.417	8.33	83.3
PTMP	0.12	9.47	3.79	4x	16.7	33.3	333.4
TTMP	0.20	15.8	4.73	0.1x	5.21	1.04	10.42
TTMP	0.20	15.8	4.73	1x	0.521	10.42	104.2
TTMP	0.20	15.8	4.73	4x	20.8	41.67	416.7

Based on this new denotation, 0.1x, 1x, and 4x concentrations of PEG_20k_-mal were also tested for aggregates (**Table 5**). After addition of PEG_20k_-maleimide to the reaction solution, the reaction solution was shaken at room temperature on a tabletop shaker at top speed for 2 h. Aggregates were then filtered using a 25 mm 0.2 μm polycarbonate track etch membrane in a Swinnex housing. The filtrate was washed three times by centrifugation (10 000 x *g* for 10 min) and resuspended in diH_2_O.

### Characterization

Hydrodynamic diameter and surface charge of nanoparticles and aggregates were characterized by dynamic light scattering (DLS) and zeta potential measurements on the ZetaPALS instrument (Brookhaven Instruments Corporation). For zeta measurements, 40 μL of nanoparticle solution was diluted to 1.2 mL with 1 mM KNO_3_ immediately prior to measurement. UV absorption spectra were taken on the Ultrospec 3000pro (GE Lifesciences). Aggregates were visualized by transmission electron microscopy (TEM). 4 μL of aggregates solution was dried onto a formvar-stabilized 200 or 300 mesh copper–carbon grid purchased from Ted Pella. TEM images were taken using FEI Tecnai 12 Twin transmission electron microscope.

### Cyanide stability

225 μL of the nanoparticle solutions in water were aliquoted into plastic cuvettes containing 225 μL phenol free RPMI 1640 complete cell growth media with 10% FBS. Serum containing media was used to prevent the salt induced coating of the plastic cuvettes[9] and to more closely mimic the surface of nanoparticles in an *in vitro* system. The particle solution was mixed with 50 μL aqueous 1M potassium cyanide (KCN) and examined by UV-Vis at 5 minutes, 1 h, 4 h, and 24 h. Warning: KCN should be used in the chemical hood, kept away from acids, and neutralized by mixing any solutions containing KCN with an excess of H_2_O_2_ in the hood.

### Cellular uptake

*24 h treatment*: 3 x 10^5^ Raw 264.7 cells (purchased from ATCC) were plated per well in a 6-well plate. After 24 h, the media was removed and replaced with 2.94 mL fresh media, and 60 μL of a solution of particles was added in treatment group per well. All particle solutions were normalized to OD = 0.438 for cell uptake experiment. Each treatment was done in triplicate. After 24 h incubation at 37°C, the media containing excess nanoparticles was removed, and the cells were washed with 1 mL of calcium and magnesium-free 1x PBS 3 times. The cells can be stored in a -20°C freezer until ready for ICP-MS analysis.

*0 h treatment*: this was included to exclude nanoparticle non-specific stickiness to cells or plate. 3 x 10^5^ Raw 264.7 cells were plated per well in a 6-well plate. After 24 h, the media was removed and replaced with 3 mL fresh media. After 24 h incubation at 37°C, the media was removed and replaced with 2.94 mL fresh media, and 60 μL of a solution of particles was added in treatment group per well. All particles were normalized to OD = 0.438 for cell uptake experiment. Immediately, excess nanoparticles was removed, and the cells were washed with 1 mL of calcium and magnesium-free 1x PBS 3 times. Each treatment was done in triplicate. The cells can be stored in a -20°C freezer until ready for ICP-MS analysis.

Notes: 1) Raw 264.7 cells in passage 3 or 4 were used in our study. Higher passages may cause decrease of cellular uptake. 2) The final cellular uptake was reported as the amount of gold measured after 24 h treatment minus the amount of gold measured at the 0 h treatment.

### Animal experiments

All animal procedures were performed in an AAALAC-accredited facility and approved by the IACUC of the City of Hope Beckman Research Institute.

*In vivo* biodistribution experiments were carried out using 8–12 weeks old female Swiss Webster (outbred) mice (Mus musculus) purchased from Charles River River (Crl:CFW(SW)). Mice were allowed to acclimate to the room for 3–7 days prior to the start of the study. Mice were designated as SPF for: Mouse rotavirus, Sendai virus, pneumonia virus of mice, mouse hepatitis virus, minute virus of mice, mouse parvovirus, Theiler murine encephalomyelitis virus, mouse reovirus type 3, mouse norovirus, lymphocytic choriomeningitis virus, mouse thymic virus, mouse adenovirus types 1 and 2, mouse cytomegalovirus, polyoma virus, K virus, ectromelia virus, Hantavirus, Prospect Hill virus, cilia-associated respiratory bacillus, *Encephalitozoon cuniculi*, and *Mycoplasma pulmonis*. Mice were also free of *Helicobacter spp*., *Clostridium piliforme*, and endo- and ectoparasites.

The mice were housed in the same room in individually ventilated cages (Optimice, Animal Care Systems, Centennial, CO) on a 12:12 light cycle. Mice were group-housed 4–5 per cage with ad libitum access to reverse-osmosis-purified water and ad libitum access to feed (LabDiet 5053, Purina Mills International, St Louis, MO). The bedding was Bed-o’Cobs 1/8 in (The Andersons, Maumee, OH), and nesting squares (Ancare, Bellmore, NY) and huts were provided for enrichment.

Nanoparticle solutions were normalized by UV-vis absorption to an optical density (OD) of 0.55. Immediately prior to injection, 30 μL of sterile 10x PBS was mixed with 270 μL nanoparticle solution to reach a final concentration of 0.5 OD and 1x PBS. 200 μL of this solution was injected intravenously (i.v.) via the tail vein, and mice were euthanized by CO_2_ at the various time points. Care was taken to ensure the various time points were spread throughout the day to account for any variability. Immediately following euthanasia, approximately 0.8–1.0 mL blood was collected via cardiac puncture, and necropsy was performed to harvest liver, spleen, kidneys, and lungs. Blood and organs were placed into pre-weighed metal-free 15 mL tubes for analysis.

### ICP-MS analysis

The total amount of gold in *in vitro* cellular uptake experiment and *in vivo* organ biodistribution experiment were both analyzed by inductively coupled plasma mass spectrometry (ICP-MS). For ICP-MS analysis, a stock solution of 2% HNO_3_/1% HCl solution was pre-made and stored in a plastic bottle. A stock solution of concentrated acid (68% HNO3/1% HCl) was made fresh. Samples from different experiments were prepared as described below. In addition, a standard curve ranging from 0.195 to 1000 ppb (1 ng mL^-1^) was made using a serial dilution of a 100 ppm (100 μg mL^-1^) gold standard (Spex Certiprep) in a 2% HNO_3_/1% HCl solution. Samples were analyzed on an Agilent 8800 ISIS (discrete sampling) in no gas mode to determine gold concentration. Rinse solution and carrier solution was 2% HNO_3_/1% HCl solution. Each sample was measured by the instrument 5 times (technical replicates). A blank solution and calibration standard were measured after approximately every 10 samples as quality control to ensure that there was no carry-over between samples, and to check for instrument consistency. The total amount of gold in each sample was calculated by multiplying the measured concentration (ppb) with the total volume of sample after dilution. %ID (injected dose) was calculated by dividing the total amount of gold in each sample by the 100% injected dose, and %ID/g was calculated by dividing the %ID by the mass (g) of each organ. Measurements below the lower limit of the standard curve were considered to be zero.

#### Cellular uptake experiment sample preparation

500 μL concentrated acid (68% HNO_3_, 1% HCl) was added into each well and allowed to digest the cell pellet at room temperature for a minimum of 15 min. The digested cell pellets were then transferred into individual 15 mL metal free tubes (SCP Science) and diluted to 5 mL with the 2% HNO_3_,1% HCl solution. The same concentration of nanoparticles that was dosed to cells (100% dose control) was also digested directly in the 15 mL tubes as a control for differences in the final amount of gold between groups. The amount of magnesium in each sample was also measured by ICP-MS for quantifying cells. A magnesium-cell standard curve was made in advance by quantifying magnesium in hemocytometer-counted number of cells.

#### Organ biodistribution experiment sample preparation

Concentrated acid (68% HNO_3_/1% HCl) was added into each tube containing blood/organs—3 mL for livers, 2 mL for blood, and 1 mL for spleen, lungs, and kidneys. The tubes were heated to 65.5°C and allowed to digest overnight until no visible particulate matter was left. Digests were diluted 10x by either transferring 1 mL of digest (liver and blood) into new tubes containing 9 mL of 2% HNO_3_/1% HCl solution, or adding 9 mL of 2% HNO_3_/1% HCl solution into the original tube (spleen, lungs, and kidneys). The same concentration of nanoparticles that was injected (100% dose control) was also digested directly in the 15 mL tubes as a control for any differences in the amount of gold between the nanoparticle samples.

### Imaging mass cytometry

Unstained, formalin-fixed and paraffin-embedded liver sections were given to the Light Microscopy Core at City of Hope for imaging. Imaging mass cytometry was performed on the Hyperion Imaging System (Fluidigm). 500 μm x 500 μm areas were imaged around the perivascular and midzonal regions. Au 197 was used to map gold distribution.

## Results

### The discrepancy between *in vitro* cellular uptake and *in vivo* biodistribution

Following our published protocols, aggregates with hydrodynamic diameter of 60 nm were synthesized by crosslinking 5 nm AuNPs with small molecule crosslinkers (pentaerythritol tetrakis(3-mercaptopropionate) (PTMP) or trimethylol tris(3-mercaptoproprionate) (TTMP)) followed by reaction termination with PEG_2K_-maleimide (PEG-mal)[5, 6]. Female Swiss Webster (outbred) mice were injected with each of these types of aggregates or HSC AuNPs or PEG AuNPs made of 50 nm AuNPs as control groups. In contrast to the marked difference between these groups for *in vitro* cell uptake, all of these materials were almost completely cleared from the blood at 1 h and the majority accumulated in the liver (**S1B Fig in S1 File**).

**Fig 1:**
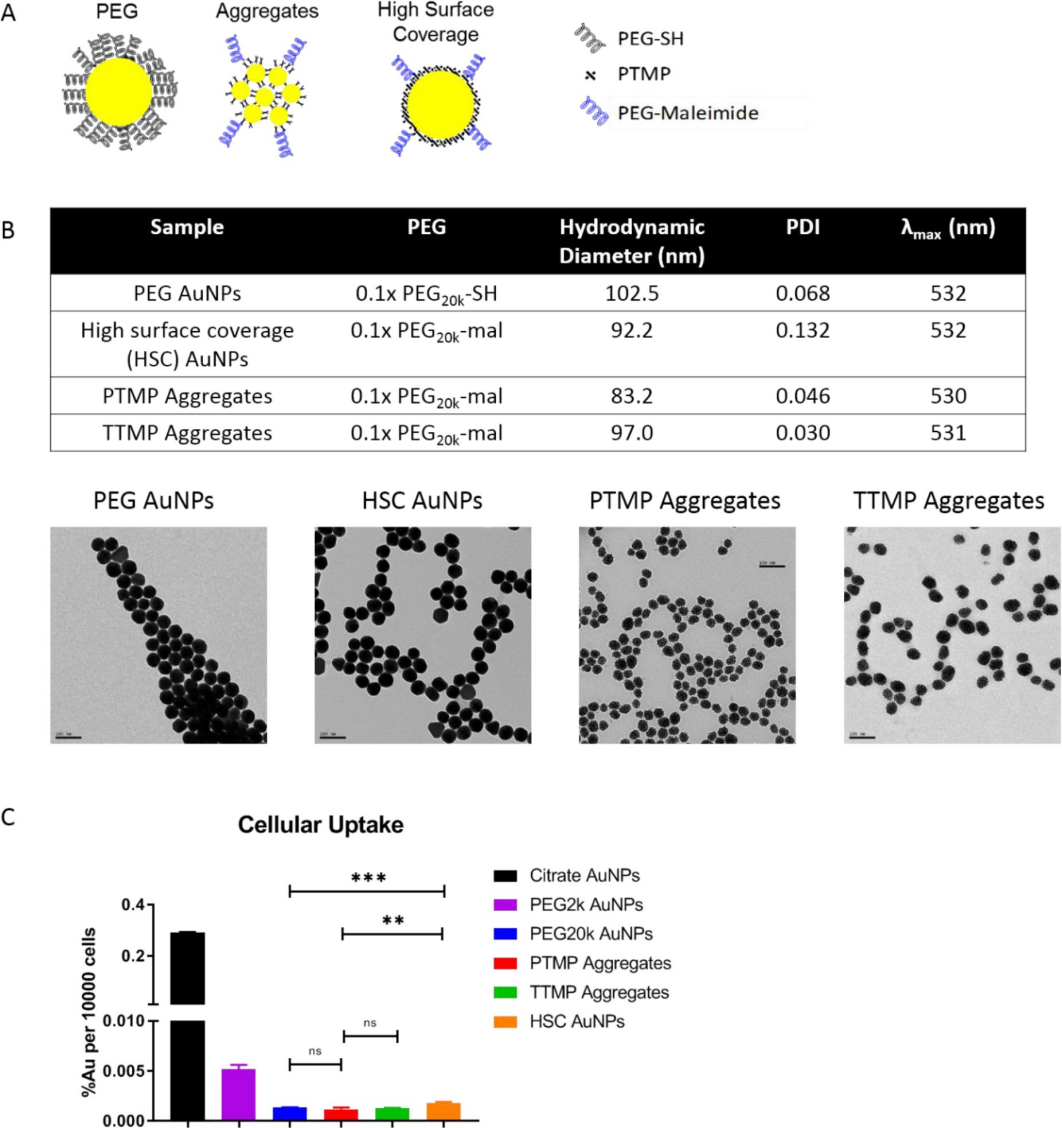
Characterization and biodistribution of different types of nanoparticles used in this study. **A**) Schematic representation of nanoparticle types used in this study. **Left**: 50 nm AuNPs coated with PEG_20k_-SH alone. **Middle**: Aggregates synthesized by crosslinking 5 nm AuNPs into larger (~50 nm) superstructures using the PTMP or TTMP crosslinkers. These aggregates were also PEGylated with PEG_20k_-mal. **Right**: 50 nm AuNPs coated with a hydrophobic small molecule crosslinker (PTMP) prior to PEGylation with PEG_20k_-mal, producing high HSC AuNPs. **B**) Characterization of nanoparticles mentioned above. Hydrodynamic diameter and PDI were measured by DLS, surface charge was measured by zeta potential, and surface plasmon resonance peak (λ_max_) was measured by UV-vis absorption. Representative TEM images were shown (Scale bar 100 nm). **C**) Cellular uptake by Raw 264.7 cells after 24 h incubation with nanoparticles. Different nanoparticle groups were normalized by OD. Results were shown as percentage of total added dosage per 10000 cells. Gold amount was measured by ICPMS.

Surprised by this, we took a step back and use 50 nm solid AuNPs as model system and systemically studied the effect of PEG coating on nanoparticle biodistribution when PEG is used in vast excess (See S1 File). These studies consisted of varying PEG length, density, and backfilling and demonstrated that when PEG is used in vast excess, using a PEG length of 10K or longer led to enhanced blood circulation time. Thus, for the aggregates and HSC AuNPs, it was evaluate if the PEGylation approach found to be optimal for solid nanoparticles (0.1x PEG_20k_) would altered their *in vitro* behavior. In the macrophage uptake experiment, PEG AuNPs, PTMP aggregates, TTMP aggregates, and HSC AuNPs with PEG_20k_ on surface were synthesized, while citrate AuNPs and PEG_2k_ AuNPs were used as positive controls (**Fig 1A**). TTMP aggregates were included because they showed even lower macrophage uptake than PTMP aggregates in a previous study[5], although they were functionalized with PEG_2k_ and had a diameter of 100 nm in that study. Here aggregates were made as metal core size ~50 nm by tuning the concentration of crosslinker and functionalized with PEG_20k_. Whether the new TTMP aggregates resulted in lower cellular uptake was re-evaluated in this study. For the characterization, PEG AuNPs, TTMP aggregates, and HSC AuNPs shared similar hydrodynamic size while PTMP aggregates were slightly smaller. They all shared low polydispersity, slightly negative surface charge, surface functionality, and spherical morphology (**Fig 1B**). High magnification TEM images were shown for the detailed cluster structure for aggregates (**S5 Fig in S1 File**). In cyanide stability assay, consistent to published findings, HSC AuNPs and aggregates capped with 0.1x PEG_20k_-mal showed no change in cyanide solution after 24 h while AuNPs capped with 0.1x PEG_20k_-SH were dissolved very fast (**S6 Fig in S1 File**). This suggested that, as we’d previously observed and reported while working with PEG_2K_, small molecule crosslinking or coating of AuNPs prior to PEGylation leads to extremely high surface coverage while direct PEGylation does not[7]. However, while the cyanide stability data for PEG_20k_ samples was very similar to PEG_2k_ samples, the difference in cellular uptake was mitigated for the PEG_20K_ samples as PEG AuNPs and both PTMP and TTMP aggregates all had similarly extremely low uptake, as compared to citrate AuNPs (**Fig 1C**). HSC AuNPs had very slightly higher uptake than PEG AuNPs and aggregates but still significantly lower than citrate AuNPs. With this relatively equivalent low uptake for all samples, we proceeded to biodistribution studies to determine if these *in vitro* findings would correlate with *in vivo* outcomes.

**Fig 2:**
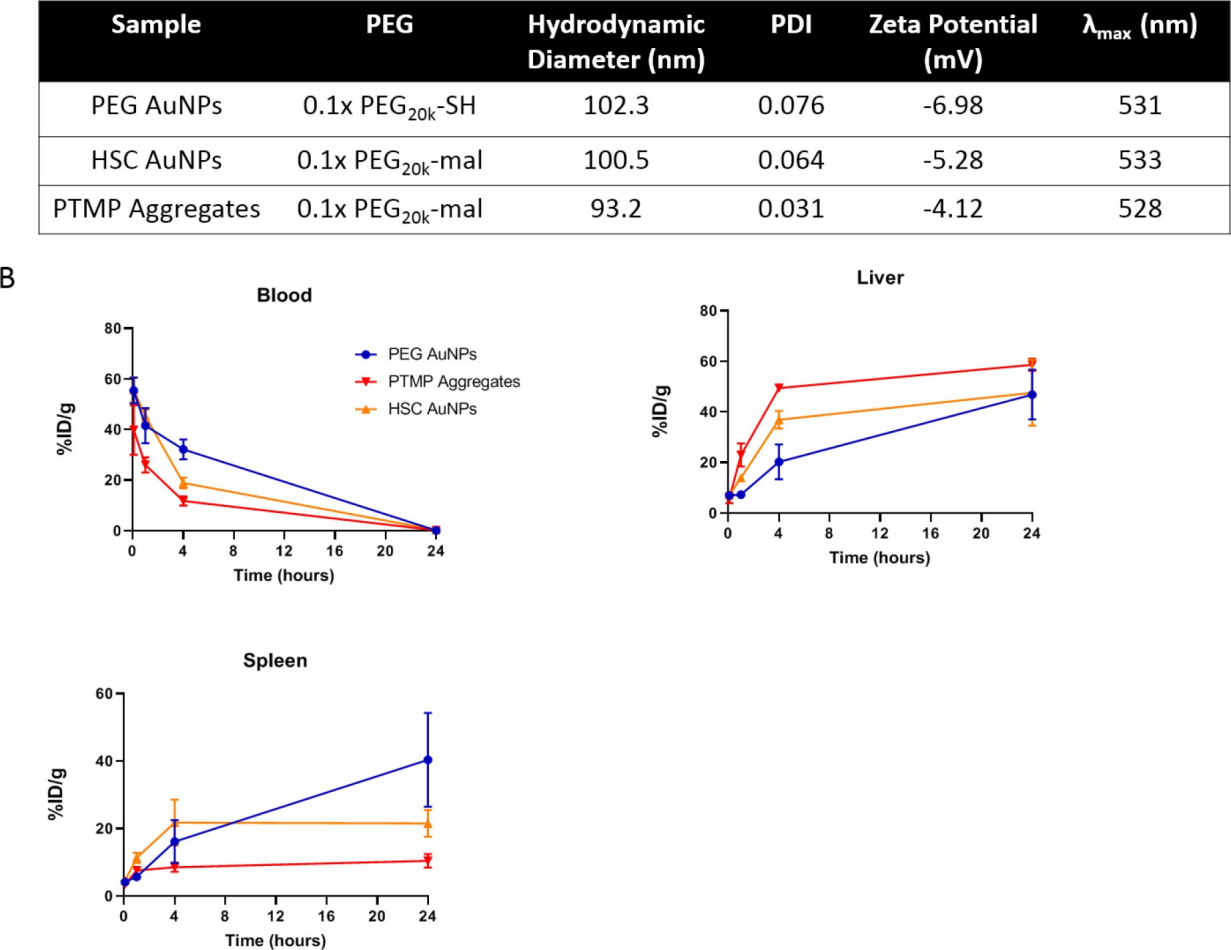
Characterization and biodistribution of PEG AuNPs, HSC AuNPs, and PTMP aggregates. **A**) Hydrodynamic diameter and PDI were measured by DLS, surface charge was measured by zeta potential, and surface plasmon resonance peak (λ_max_) was measured by UV-vis absorption. **B**) Amount of gold measured by ICP-MS at various time points in the blood, liver, and spleen, respectively, reported as percent injected dose per gram of tissue. Error bars represent standard deviation, n = 3.

In one pilot biodistribution experiment, control PEG AuNPs, HSC AuNPs, and PTMP aggregates were chosen to represent three different types of materials. Particles were administrated via tail vein and the blood, liver, and spleen were collected at 5 min, 1 h, 4 h, and 24 h. Surprisingly, HSC AuNPs and PTMP aggregates were cleared from the circulation system and accumulated in the liver significantly faster than PEG AuNPs (**Fig 2B**). Furthermore, while HSC AuNPs showed slightly higher cellular uptake than aggregates (**Fig 1C**), they circulated longer in the blood and accumulated less in the liver than aggregates (**Fig 2B**). It’s interesting that compared to the other two groups, PTMP aggregates presented higher accumulation in the liver but lower accumulation in the spleen. In addition, HSC AuNPs accumulated in the spleen faster than PEG AuNPs within 4 h, same as in the liver; however, while they had same accumulation in the liver after 24 h, HSC AuNPs showed lower accumulation than PEG AuNPs in the spleen. It has been reported that size and surface charge of nanoparticles can redistribute the organ accumulation[10]. However, these parameters cannot explain the difference of biodistribution here since those particles share very similar size and surface charge. The mechanisms of liver and spleen accumulation of nanoparticles needs further investigation.

**Fig 3:**
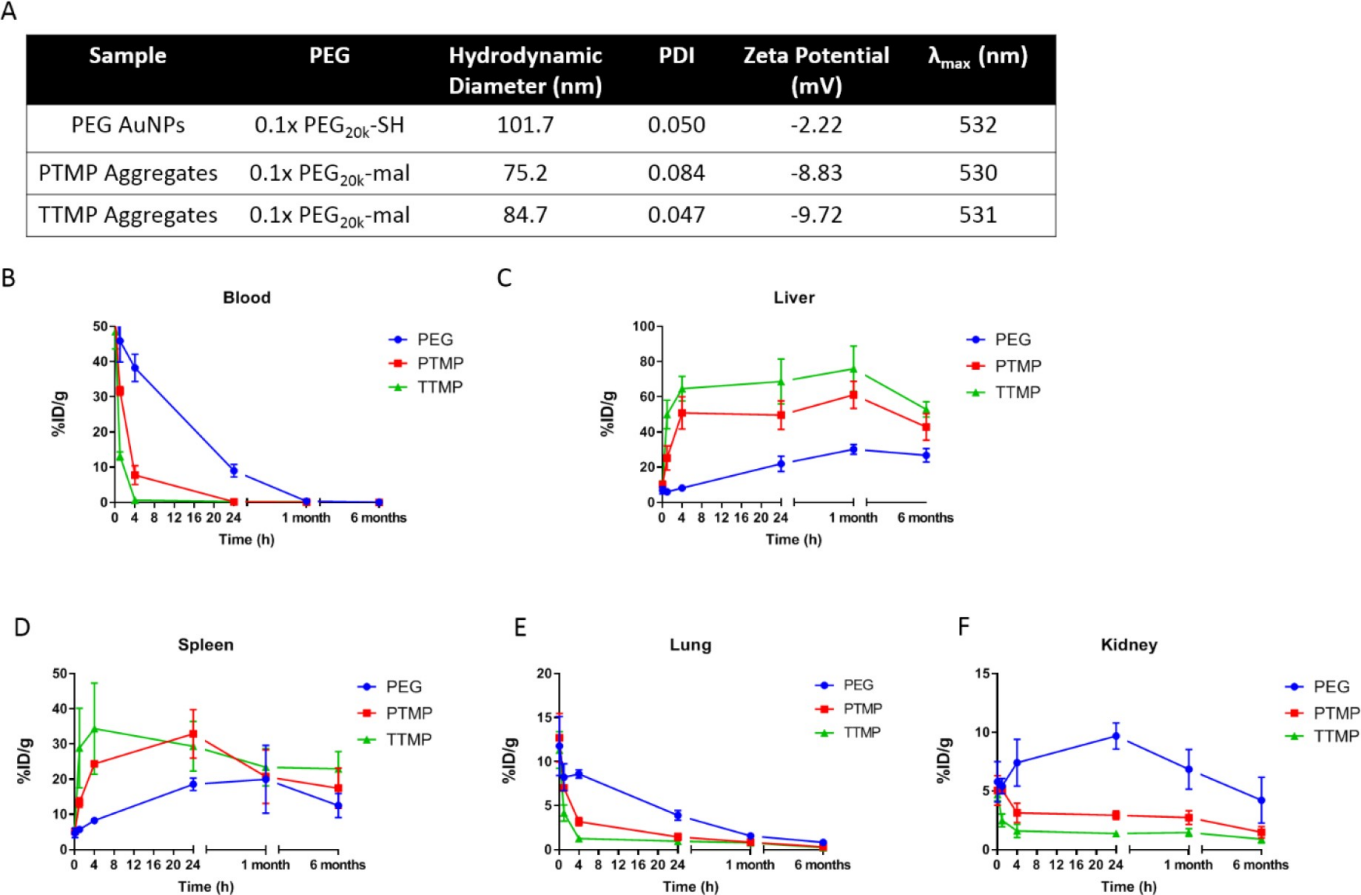
Characterization and long-term biodistribution of PEG AuNPs, PTMP aggregates, and TTMP aggregates. **A**) Hydrodynamic diameter and PDI were measured by DLS, surface charge was measured by zeta potential, and surface plasmon resonance peak (λ_max_) was measured by UV-vis absorption. **B-F**) Amount of gold measured by ICP-MS at various time points in the blood, liver, spleen, lungs, and kidneys, respectively, reported as percent injected dose per gram of tissue.

In the above pilot study, it is most surprising to us that aggregates were cleared much faster from the blood than control PEG AuNPs. To probe this result, we performed another biodistribution experiment with PEG AuNPs and both aggregates prepared using PTMP and TTMP. Long-term time points were also added for the purpose of monitoring long-term clearance out of the liver. We hypothesized that aggregates would have enhanced clearance from the liver compared to solid PEG AuNPs. This is because aggregates were crosslinked with small molecule linkers which can potentially be metabolized, releasing the smaller subunit nanoparticles which could have more rapid clearance than the larger parent structure. Two previous reports on aggregates assembled from 5 nm gold nanoparticles, either using DNA-crosslinkers[11] or non-covalent liposomal assembly[12], found that after these aggregates are accumulated in the liver the clearance is extremely slow for the 5 nm subunits. For our study, the blood, liver, spleen, lung, and kidneys were collected at 5 min, 1 h, 4 h, 24 h, 1 month, and 6 months post-injection and gold was quantified by ICP-MS. Again, unexpectedly but consistently, the short-term biodistribution (<24 h) results showed that both PTMP and TTMP aggregates had significantly shorter blood circulation time and higher accumulation in the liver compared to PEGylated solid AuNPs (**Fig 3B–3C**). TTMP aggregates also performed worse than PTMP aggregates. Aggregates had slightly smaller size than PEG AuNPs, but this is unlikely to explain why they had worse circulation. Interestingly, the process of liver accumulation lasted for up to a month for all particles although the majority was accumulated within the first 4 h (**Fig 3C**). The trend of lung accumulation matched the blood, most likely due to the residual blood in the lungs since the mice were not perfused prior to organ collection (**Fig 3E**). It is also interesting that the timelines of PEG AuNPs and aggregate accumulation in the kidneys are different. PEG AuNPs accumulated to a high level within a day but also quickly started decreasing after 24 h. However, for aggregates, the accumulation was modest compared to PEG AuNPs and clearance started only after 1 month. (**Fig 3F**).

**Fig 4:**
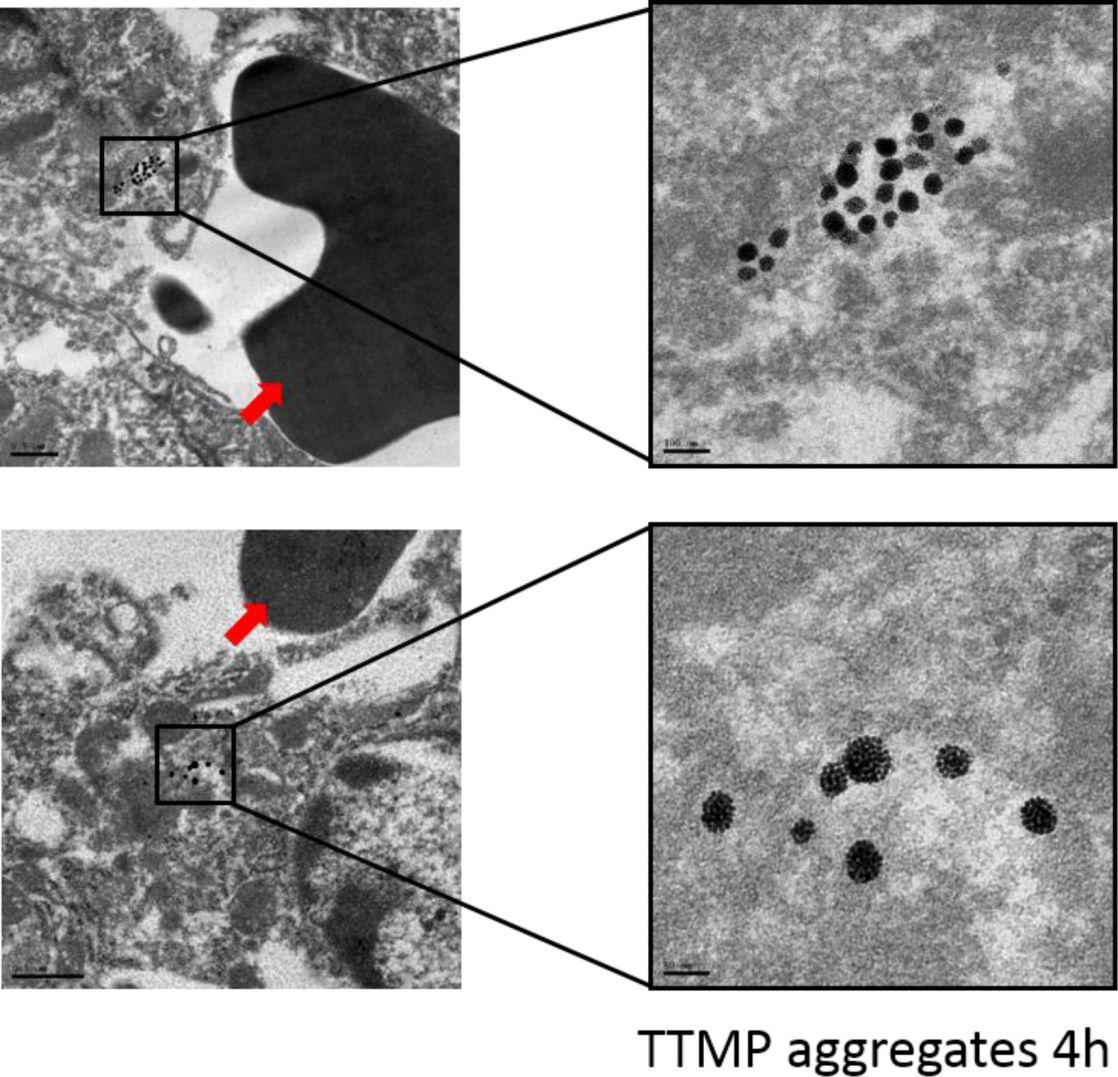
Representative TEM images of liver sections at the 4 h time point post-injection of TTMP aggregates. Aggregates were only found in perivascular areas and were not seen in extracellular space. Red arrow indicated red blood cells.

In our long-term clearance study, there were slight decreases in the liver after 6 months for all groups, indicating slight clearance. Although, it seems aggregates were cleared a little faster than solid AuNPs. Specifically, between the 1 month and 6 month time point, 29.8% PTMP aggregates and 30.3% TTMP aggregates were cleared from the liver while only 11.3% PEG AuNPs were cleared. Nevertheless, whether the faster clearance was due to dissociation of aggregates in the liver needs to be further studied. TEM images of liver sections (4 h time point) identified intact aggregates within liver cells (**Fig 4**) but this does not rule out some dissociation into subunit particles, as individual 5 nm AuNPs are difficult to visualize by TEM. That being said, so far there is no direct evidence supporting the aggregates were dissociable in the liver. One interesting finding was that PEG AuNPs start decreasing in the kidneys from 24 h (**Fig 3F**). There were no published reports indicating that nanoparticles as large as 50 nm (core size) could be cleared from the kidneys through the renal system. For aggregates, there were also decrease in the kidneys after 1 month. However, the mechanism of how the particles got cleared from the kidneys needs further investigation.

Taken together, the biodistribution result showed the discrepancy between *in vitro* and *in vivo* outcomes: PTMP and TTMP aggregates have significantly shorter blood circulation time and higher liver accumulation *in vivo* compared to solid PEG AuNPs, regardless of higher surface coverage and similar cell uptake *in vitro*. Aggregates also accumulated faster in the liver than HSC AuNPs, regardless of similar surface coverage and slightly lower cellular uptake. Thus, neither surface coverage nor cellular uptake is a good predictor of biodistribution.

#### Suborgan distribution in the liver

**Fig 5:**
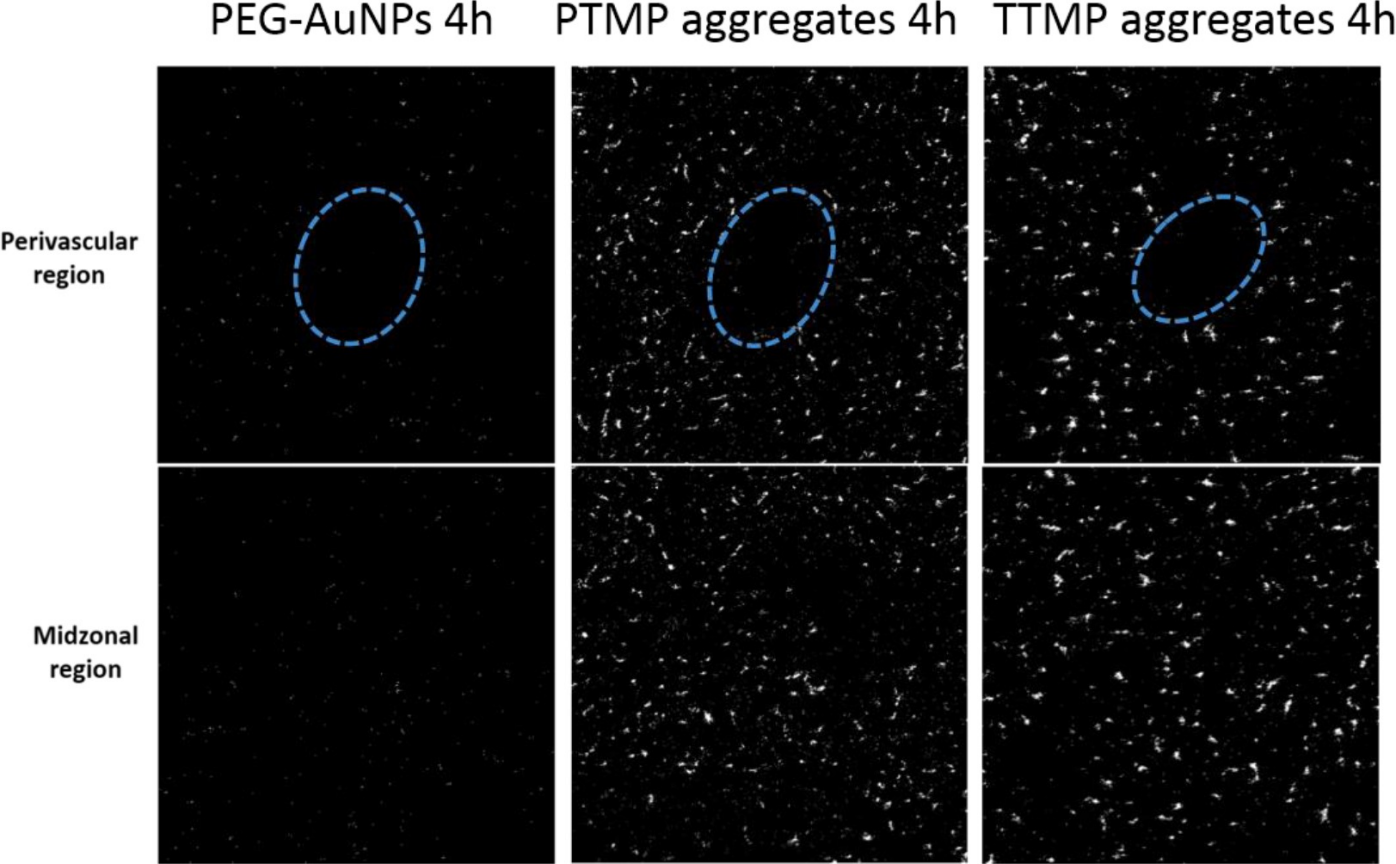
Imaging mass cytometry images of liver sections (500 μm x 500 μm) at the 4 h time point. Gold distribution (white dots) in either a perivascular (top) or midzonal (bottom) region were shown. The blue circle indicated the locations of vessels. PBS control images were completely black (S10 Fig in S1 File).

In part due to the discrepancy seen between *in vitro* cellular uptake and *in vivo* biodistribution, we wanted to investigate the location of nanoparticle accumulation in the liver as it has been reported that liver microarchitecture plays a big role in nanoparticle accumulation[13]. The authors hypothesized that because blood flows from portal vein to central vein, high concentration of nanoparticles will interact with cells in periportal zone first which provides a chance for cellular uptake. Supporting this, they found that nanoparticles preferentially accumulated in the portal triad zone relative to the central vein zone. To determine whether aggregates and solid AuNPs showed a similar distribution in our studies, we first used imaging mass cytometry to determine the spatial distribution of gold within the liver architecture. We chose the liver sections collected at the 4 h time point from the biodistribution experiment in **Fig 3**because this time point had the biggest difference in accumulation levels between groups. For each group, a perivascular region (portal and central areas not differentiated) and a midzonal region were imaged. PBS control mice liver slice was used as control which was completely black showing no gold signal. The signal threshold was kept consistent among groups. However, gold signals in all groups were uniformly distributed, indicating no perivascular (portal area) distribution (**Fig 5**). Since the gold signals were punctate, we hypothesized that the particles co-accumulated inside the cells or inside the sinusoidal spaces. Liver tissue samples were imaged by TEM because it can give us the most detailed structures. TTMP aggregates (4 h) was chosen as an example due to its highest accumulation. Also, PEG AuNPs were hard to be differentiated from the liver tissue background (containing a lot of dark dots from staining process) but aggregates could be differentiated due to the unique cluster structure. As shown in **Fig 4**, aggregates were always found as a group inside a cell but not extracellular space. The cell type containing aggregates was not clear but it was always next to vasculature because red blood cells can be seen in the gap space.

#### Further optimization of PEGylation on nanoaggregates

It was unexpected that aggregates had such reduced blood circulation as compared to solid AuNPs, despite sharing very similar physicochemical properties. As indicated by cyanide stability results, the surface of gold in the aggregates was fully coated by small crosslinkers which increase the accessible surface area than the initial metallic surface area due to the multiple thiols on each crosslinker. When PEG was beyond enough in 50 nm AuNP model system, it is possible it did not cap enough surface in aggregates. To test this, PTMP and TTMP aggregates were capped with increasing concentrations of PEG_20k_-mal (0.1x, 1x, 4x) (**Table 5**). Compared to solid AuNPs with 0.1x PEG_20k_-SH, aggregates with different concentrations of PEG_20k_-mal showed no significant differences in hydrodynamic diameter or surface charge (**S7A Fig in S1 File**). As expected, all aggregates were very stable in cyanide compared to PEG AuNPs (**S6B Fig in S1 File**). However, the 1 h biodistribution experiment showed that the concentration of PEG_20k_-mal in the range we tested did not significantly change the biodistribution (**S7C-S7G Fig in S1 File**). However, it is possible that excessive long-chain PEG_20k_-mal cannot access buried thiols due to steric hindrance. Thus, we also used shorter PEG_750_ or small maleimides which have less steric hindrance to backfill the aggregates. PTMP aggregates were first capped with 0.1x PEG_20k_-mal and then backfilled with 1x concentration of PEG_750_ or maleimides (1.1x maleimide:thiol), respectively (**S8A Fig in S1 File**). PEG AuNPs and PTMP aggregates with or without different backfilling showed similar hydrodynamic diameters and surface charge (**S8B Fig in S1 File**). This time, in the biodistribution experiment, the blood, liver, spleen, lungs, and kidneys were collected at 4 h post-injection due to larger difference than 1 h as seen in previous time-course biodistribution experiment. Consistent with previous results (**Figs 2 and 3**), at 4 h time point, PTMP aggregates had lower concentration in the blood compared to PEG AuNPs, and inversely higher concentration in the liver. However, again, backfilling with either PEG_750_-mal or maleimide did not help the aggregate circulation in the blood (**S8C Fig in S1 File**). In fact, backfilling with maleimide actually accelerated the clearance from the blood.

#### The role of fasting in nanoparticle biodistribution

In the systemic biodistribution studies, we were surprised by the magnitude of variance in treatment groups. For instance, at 1 h time point as shown in **Figs 1 and 3**, we always found the level of Au in one mouse blood was either much higher or lower than the other two mice and this correlated with the inverse result for liver accumulation (i.e. A mouse with unusually high amount in the blood would have an unusually low amount in the liver). One parameter we considered was whether how recently the mice had eaten could affect the biodistribution of nanoparticles. To normalize, mice were fasted before the injection of nanoparticles. Six mice were included for larger sample amount. Mice were fasted for 16 h before administration of 0.1x PEG_20k_-SH PEG AuNPs and continued fasting for 4 h post-injection until the blood, liver, spleen, lung, and kidneys were collected. The absolute amount of Au accumulated at different organs were similar as previous biodistribution data at 4 h time points. However, one outlier was still seen in 6 mice which indicated that the variance of results was not caused by food (**S9 Fig in S1 File**). It is possible that simply the heterogeneity of the outbred Swiss Webster mice or their immune status could be responsible for the variance, but further pursuit of this question lies outside the current manuscript.

## Discussion

In this work, we have compared the blood half-lives and biodistribution of several gold nanomaterials–namely PEGylated solid AuNPs, solid AuNPs coated with a small molecule layer and then PEGylated, and nanoaggregates assembled from 5 nm AuNPs. All of these materials had similar physiochemical properties, but displayed very different *in vitro* and *in vivo* behavior and there was limited correlation between the *in vitro* and *in vivo* results. We began this investigation due to the remarkably lower uptake by macrophages of our nanoaggregates coated with PEG_2K_ as compared to solid AuNPs also coated with PEG_2K_. However, *in vivo* all of the materials cleared quite rapidly. Solid AuNPs were used to probe the effect of PEG length and we found that increasing the molecular weight of PEG from 2k to 10k greatly increases blood circulation time of 50 nm AuNPs; further increase to 20k did not make further improvement. The concentration of PEG we used in this study (0.1x PEG_20k_ in our nomenclature) was also above the saturation density. Backfilling with short PEG beyond did not further improve the blood circulation. Although while working with PEG_2k_, surface coverage is inversely correlated to cellular uptake[7], surface coverage cannot predict uptake when using PEG_20k_ as PEG AuNPs and aggregates had equivalent low cellular uptake despite the PEG AuNPs being much more rapidly dissolved by cyanide. When moving to *in vivo* study, our aggregates had faster clearance from the blood and accumulation in the liver than the PEG AuNPs. It is still unclear what parameter leads to the difference between PEG AuNPs and aggregate biodistribution. We think it is unlikely to be caused by uncapped surface free thiols because neither increasing PEG_20k_ input nor backfilling aggregates with maleimide or shorter PEG_750_ improved the blood circulation time or decreased liver accumulation. Another potential factor is the different conjugation chemistry, as the PEG is conjugated directly to the solid AuNPs via a Au-S bond, while the PEG is conjugated to the aggregates via a S-maleimide reaction. It is known that thiol-gold interaction is more stable than thiol-maleimide conjugation. Thus, it is possible that thiol-maleimide bond was disrupted *in vivo* and aggregates were quickly cleared out from blood after losing PEG coating. Further studies are needed to investigate the stability and metabolism of PEG coatings on nanoparticles *in vivo*.

The dogma within the field of nanomedicines is that blood clearance of nanoparticles is driven by cellular uptake by phagocytic cells such as Kupffer cells in the liver[3, 4], and thus, particle formulations with decreased macrophage uptake *in vitro* will have improved blood circulation time *in vivo*[14–19]. While there is a correlation between reduced *in vitro* uptake and *in vivo* circulation for some particles, for the gold nanoparticles studied here there was little to no predictive value for the *in vitro* models. This strongly suggests that for nanoparticles to have a greater impact in translational research, there is an urgent need to develop better *in vitro* predictive tools, such as using primary cell platforms to better mimic physiological condition or using organ-on-a-chip system for better organ microarchitecture/fluid flow mimicking. Alternatively, it may be actually most efficient to use *in vivo* models as the primary discovery format.

## Supporting information

S1 File (DOCX)
