# Supplementary material for: A Systematic comparison of in vitro cell uptake and in vivo biodistribution for three classes of gold nanoparticles with saturated PEG coatings
Source: PLoS One. 2020 Jul 2;15(7):e0234916. doi: 10.1371/journal.pone.0234916 (PMC7332061; doi:10.1371/journal.pone.0234916)
Supplement: S1 File — (DOCX) [file pone.0234916.s001.docx]

Supporting Information For:

A Systematic Comparison Of *In Vitro* Cell Uptake And *In Vivo* Biodistribution For Three Classes Of Gold Nanoparticles With Saturated PEG Coatings

Yijia Zhang^¶^, Alice T. Liu^¶^, Yvonne Cornejo, Desiree Van Haute, Jacob M. Berlin^*^

Department of Molecular Medicine, Irell and Manella Graduate School of Biological Sciences, Beckman Research Institute, City of Hope National Medical Center, Duarte, California 91010, United States

^*^ Corresponding author

E-mail: jacobberlincoh@gmail.com

*^¶^*These authors contributed equally to this work.

## Systematic optimization of PEGylation on solid AuNP model system

### PEG length

The reported effect of PEG length on nanoparticle biodistribution is contradictory. It has been reported that increasing PEG length from 2k to 10k on 20-100 nm AuNPs significantly increases blood half-life([1](#_ENREF_1)). However, for other materials, PEG did not have significant effect([2](#_ENREF_2)). Thus, we first evaluated the impact of PEG length in our system by measuring the biodistribution of 50 nm AuNPs PEGylated with PEG-SH at molecular weight 2k, 5k, 10k, and 20k. The concentration of PEG-SH used was such that there was an equivalent concentration of thiols as was used to synthesize PTMP aggregates (aggregate size: ~ 50 nm metallic core). For characterization, hydrodynamic diameter increased as PEG length increased (**S2A Fig**). The surface coverage of particles was measured by the stability in cyanide solution ([3](#_ENREF_3), [4](#_ENREF_4)). Cyanide stability assay showed that AuNPs with PEG of all lengths were dissolved within 20 minutes at a comparable scale to citrate AuNPs, indicating no significant difference in surface coverage (**S2B Fig**). Then a single time point biodistribution experiment was performed where the blood and liver were collected 1 h after injection (n=3). It was found that increasing PEG length from 2k to 10k significantly elongated blood circulation time and reduced liver accumulation([1](#_ENREF_1)) and that 20k performed similarly to 10k **(S2C Fig)**. Here PEG_20k_ was chosen for later studies because there appeared to be a trend towards lower liver accumulation for PEG_20k_ as compared to PEG_10k_.

### PEG density

As another important factor, PEG density on the surface of nanoparticles has also been reported to influence pharmacokinetics and biodistribution of polymer and lipid nanoparticles ([5-7](#_ENREF_5)). To test this parameter in our system, we PEGylated 50 nm AuNPs at four different concentrations of PEG_20k_-SH—0.005x, 0.1x, 1x, and 10x, equivalent to approximately 6.5, 129, 1292, and 12925 PEG added per nm^2^, respectively. All concentrations tested were higher than the reported saturation density of PEG_20k_-SH (0.14-0.21 PEG per square nm([8](#_ENREF_8))), but we decided to use these high concentrations of PEG in order for the concentrations of PEG-SH used here to be equivalent to the concentrations of PEG-maleimide used to cap thiols on the aggregates. The PEG-maleimide concentrations are, relatively speaking, so high because they were chosen to ensure capping of all possible free thiols in the aggregates which are more abundant than the initial metallic surface area due to the multiple thiols on each crosslinker. AuNPs capped with different PEG concentrations did not show significant difference in hydrodynamic diameter, polydispersity, or surface charge (**S3A** **Fig**) and surface coverage (**S3B Fig**). In a time-course biodistribution study, the blood, liver, and spleen were collected at 5 min, 1 h, 4 h, and 24 h post treatment (n=3) (**S3C Fig**). For comparison, gold detected in the blood and liver of 1 h time point was shown in **S3D Fig**. PEG concentrations in the range we tested did not show significant difference in the blood circulation time and liver accumulation, which suggests that the PEG concentrations used in AuNP-aggregates studies are well beyond the concentration required to saturate the surface.

### PEG backfilling

A previous study had also shown that backfilling with low molecular weight PEG on the surface of nanoparticles can reduce protein adsorption and decrease non-specific nanoparticle-cell interactions *in vitro*([9](#_ENREF_9)). Thus, we tested whether backfilling with short PEG could further improve the particle blood half-life. 50 nm AuNPs were first PEGylated with PEG_20k_-SH at two different concentration- 0.1x and 1x, (equivalent to approximately 129 and 1292 PEG added per nm^2^, respectively), and then immediately backfilled with PEG_1k_-SH at 0.005x, 1x, and 10x concentrations (equivalent to approximately 6.5, 1292, and 12925 PEG added per nm^2^, respectively). For particle characterization, the surface charge or hydrodynamic diameter were similar with or without backfilling with PEG_1k_-SH (**S4A Fig**). Cyanide stability assay also did not show significance change when particles were backfilled with PEG_1k_-SH; there may be a slight trend for increased PEG concentration causing slightly slower dissolution in cyanide, but all particles were dissolved quickly within 20 min (**S4B Fig**). As shown in the 1 h biodistribution experiment, the particles backfilled with PEG_1k_-SH did not show significant higher concentration in the blood compared to the one without backfilling (**S4C Fig**). Interestingly, while showing no difference in the blood clearance, higher concentration of PEG (including all 1x PEG_20k_-SH and 0.1x PEG_20k_-SH backfilled with 10x PEG_1k_-SH) showed higher liver accumulation. Collectively, the results suggested no need of backfilling to maximize blood circulation *in vivo* in our system. The concentration of 0.1x PEG_20k_ without backfilling with short PEG was chosen to be used in further studies.

**
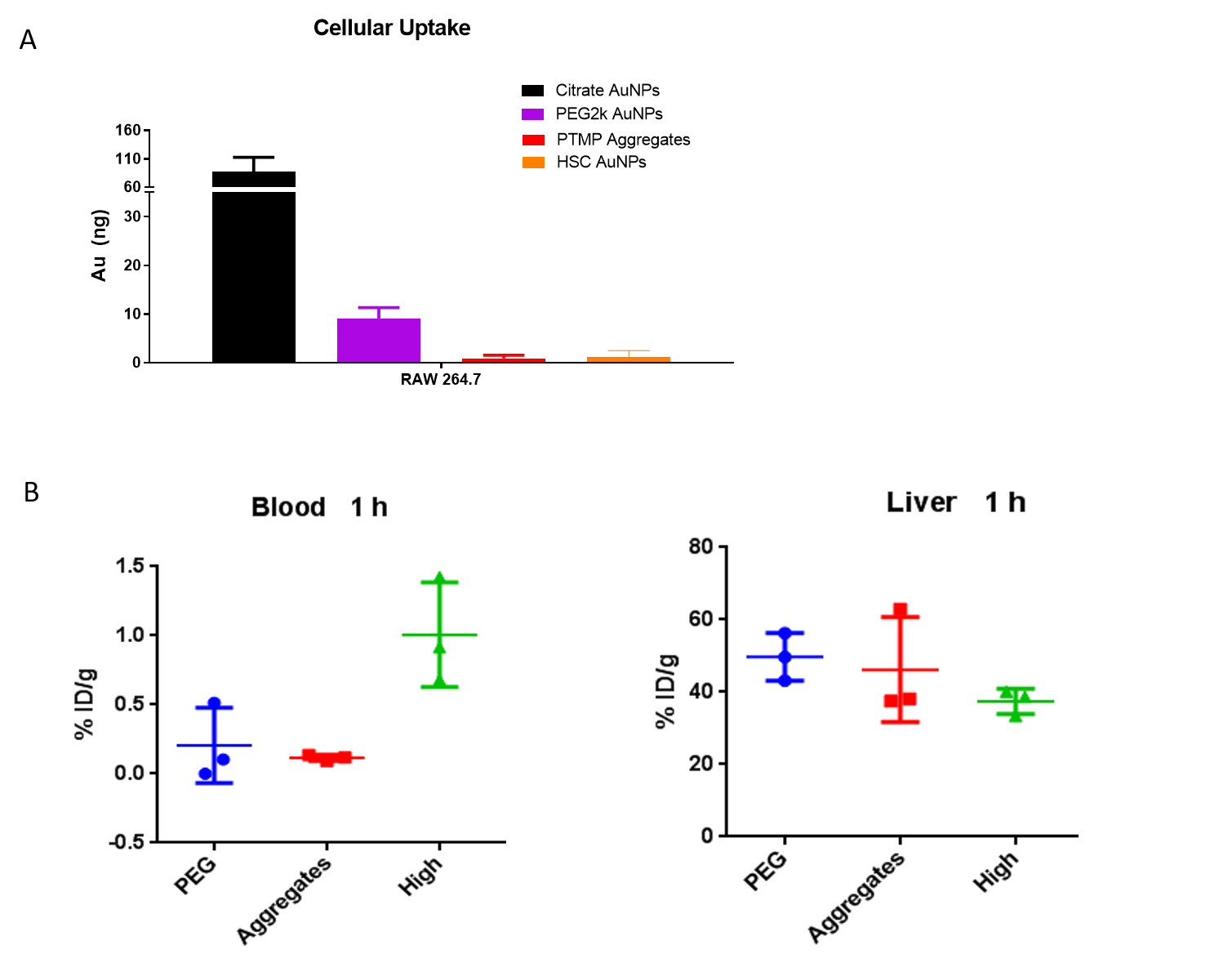
S1 Fig. Pilot study of *in vitro/in vivo* correlation.** A) Cellular uptake of citrate AuNPs, PEG AuNPs, PTMP aggregates, and HSC AuNPs. All particles except citrate AuNPs were coated with PEG_2k_. Raw 264.7 cells treated with 3.6e8 nanoparticles/mL were analyzed by inductively coupled plasma mass spectrometry for gold content at 24 h. Each bar is the average of three independent experiments each with four replicates; error bars are the standard error mean. Image adapted from Van Haute *et al*, *ACS Nano*, 2018. B) Amount of gold measured by ICP-MS at 1 h post-injection in the blood and liver, respectively, reported as percent injected dose per gram of blood. Error bars represent standard deviation, n=3.

**
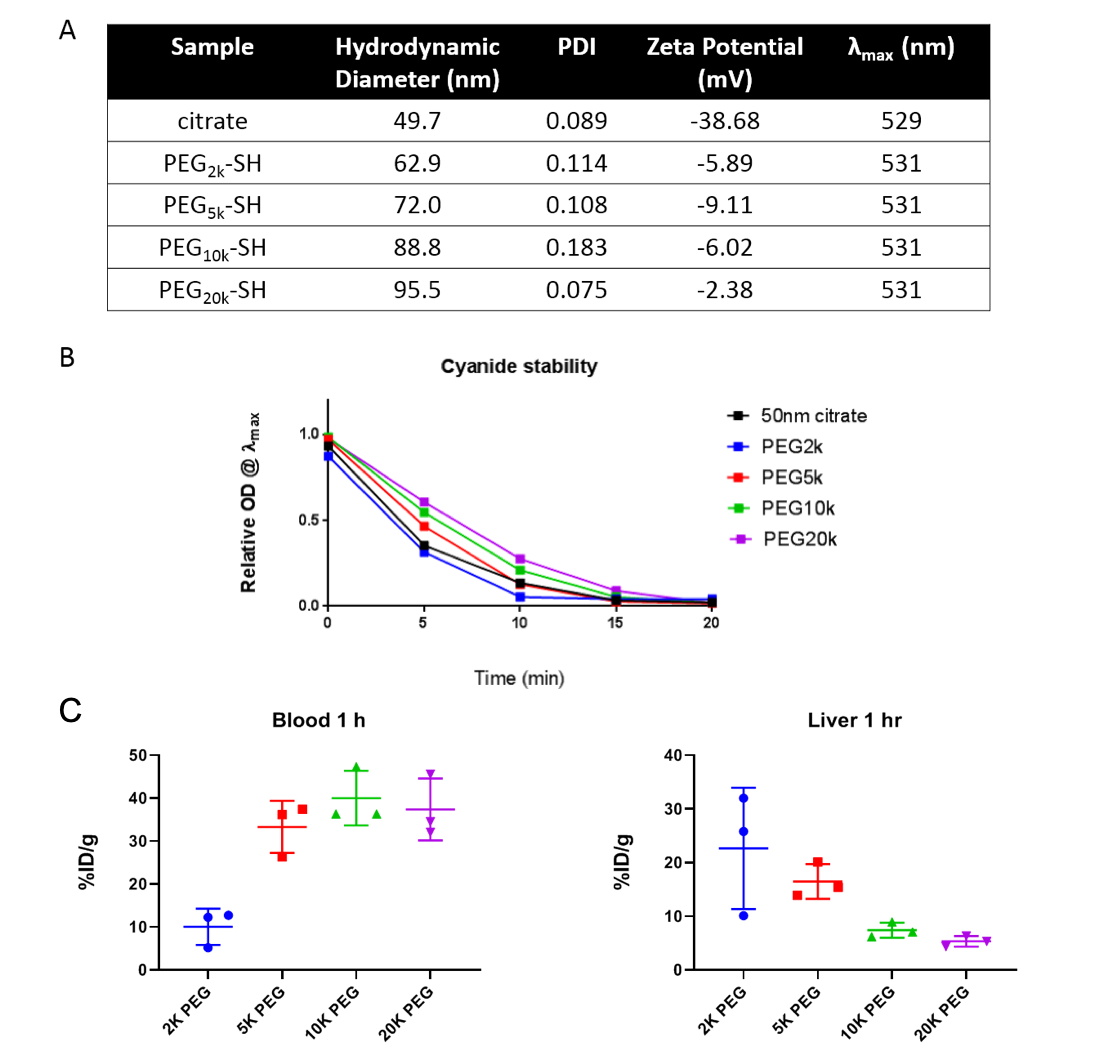
**

**S2 Fig. Characterization and biodistribution of 50 nm AuNPs functionalized with PEG-SH of increasing molecular weight (2k, 5k, 10k, and 20k).** **A**) Hydrodynamic diameter and PDI were measured by DLS, surface charge was measured by zeta potential, and surface plasmon resonance peak (λ_max_) was measured by UV-vis absorption. **B**) Amount of gold measured by ICP-MS at 1 h post-injection in the blood and liver, respectively, reported as percent injected dose per gram of blood. Error bars represent standard deviation, n=3. **C**) Cyanide stability of particles. The relative decrease in optical density (OD) at λ_max_ of particles in 0.1M cyanide was measured by UV-Vis spectroscopy.


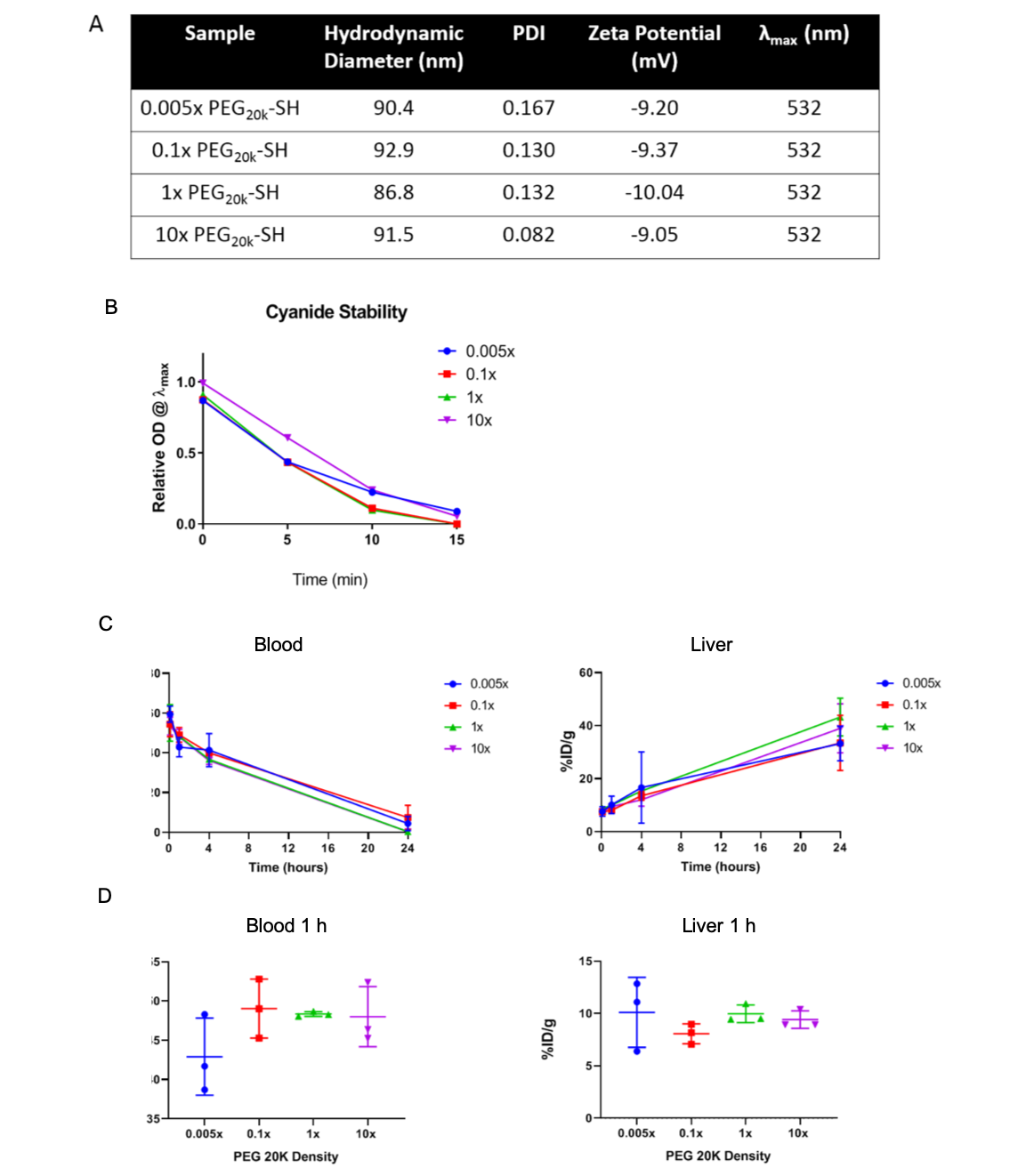


**S3 Fig. Characterization and biodistribution of 50 nm AuNPs functionalized with varying concentrations of PEG_20k_-SH (0.005x, 0.1x, 1x, 10x). A**) Hydrodynamic diameter and PDI were measured by DLS, surface charge was measured by zeta potential, and surface plasmon resonance peak (λ_max_) was measured by UV-vis absorption. **B**) Amount of gold measured by ICP-MS at time-course in the blood and liver, respectively, **C**) Amount of gold measured by ICP-MS at 1 h post-injection in the blood and liver, respectively, reported as percent injected dose per gram of blood. Error bars represent standard deviation, n=3. **D**) Cyanide stability of particles. The relative decrease in optical density (OD) at λmax of particles in 0.1M cyanide was measured by UV-Vis spectroscopy.

**
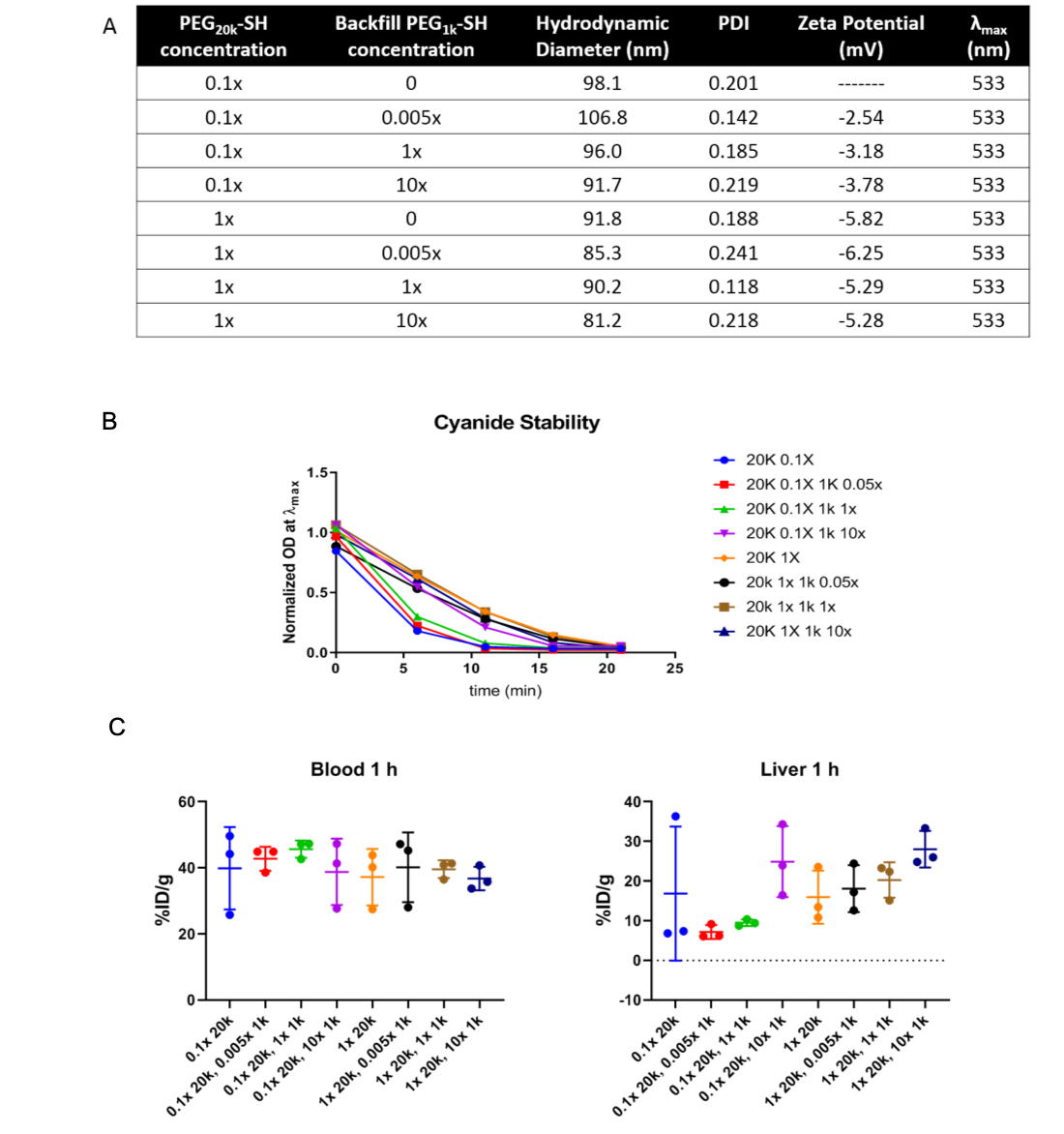
**

**S4 Fig. Characterization and biodistribution of 50 nm AuNPs functionalized with PEG_20k_-SH and backfilled with PEG_1k_-SH at varying concentrations. A**) Hydrodynamic diameter and PDI were measured by DLS, surface charge was measured by zeta potential, and surface plasmon resonance peak (λ_max_) was measured by UV-vis absorption. **B**) Amount of gold measured by ICP-MS at 1 h post-injection in the blood and liver, respectively, reported as percent injected dose per gram of blood. Error bars represent standard deviation, n=3. **C**) Cyanide stability of particles. The relative decrease in optical density (OD) at λmax of particles in 0.1M cyanide was measured by UV-Vis spectroscopy.

**
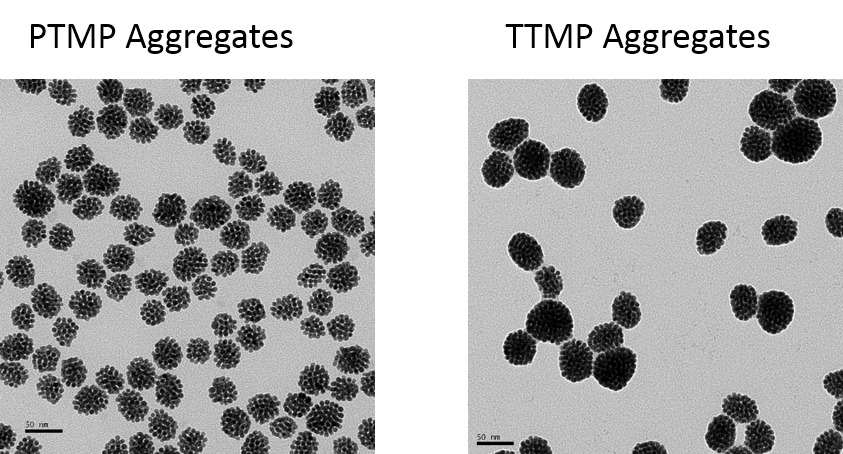
**

**S5 Fig. Representative TEM images of PTMP and TTMP aggregates (High magnification).**

**
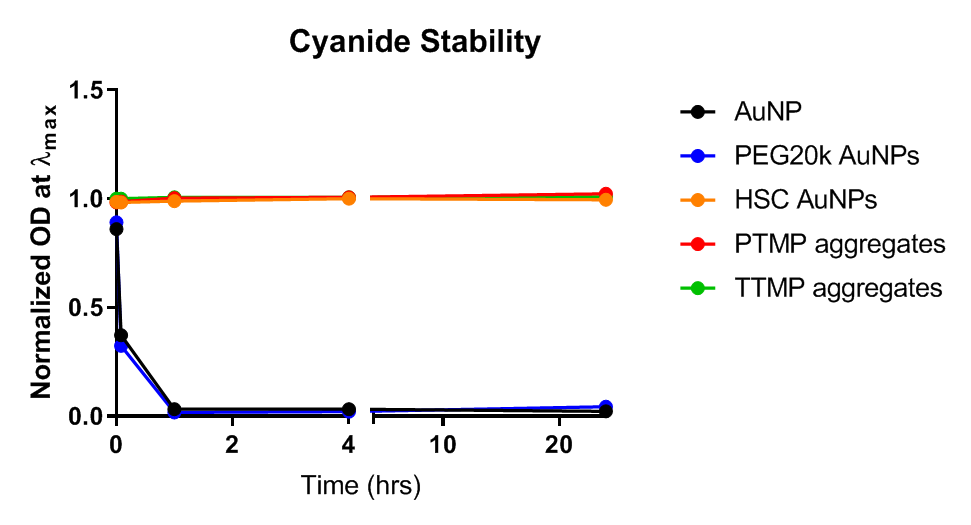
**

**S6 Fig. Cyanide stability of particles.** The relative decrease in optical density (OD) at λmax of particles in 0.1M cyanide was measured by UV-Vis spectroscopy.

**
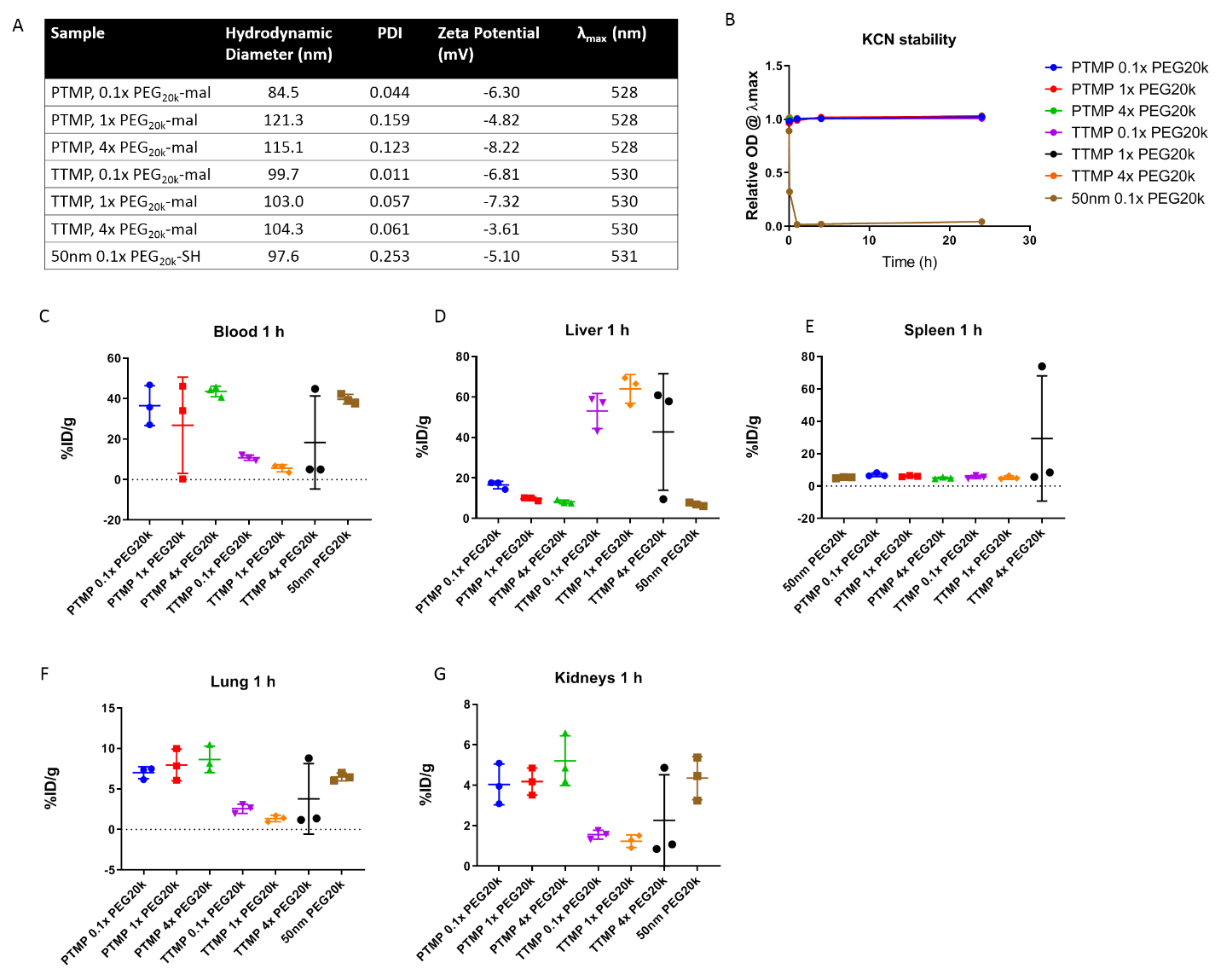
S7 Fig. Characterization and biodistribution of PTMP and TTMP aggregates functionalized with varying concentrations of PEG_20k_-mal.** A) Hydrodynamic diameter and PDI were measured by DLS, surface charge was measured by zeta potential, and surface plasmon resonance peak (λ_max_) was measured by UV-vis absorption. B) Stability of particles in 0.1M cyanide, as measured by the relative decrease in optical density (OD) at λ_max_ over time. C-G) Amount of gold measured by ICP-MS at 1 h post-injection in the blood, liver, spleen, lungs, and kidneys, respectively, reported as percent injected dose per gram of tissue. Error bars represent standard deviation, n=3.

**
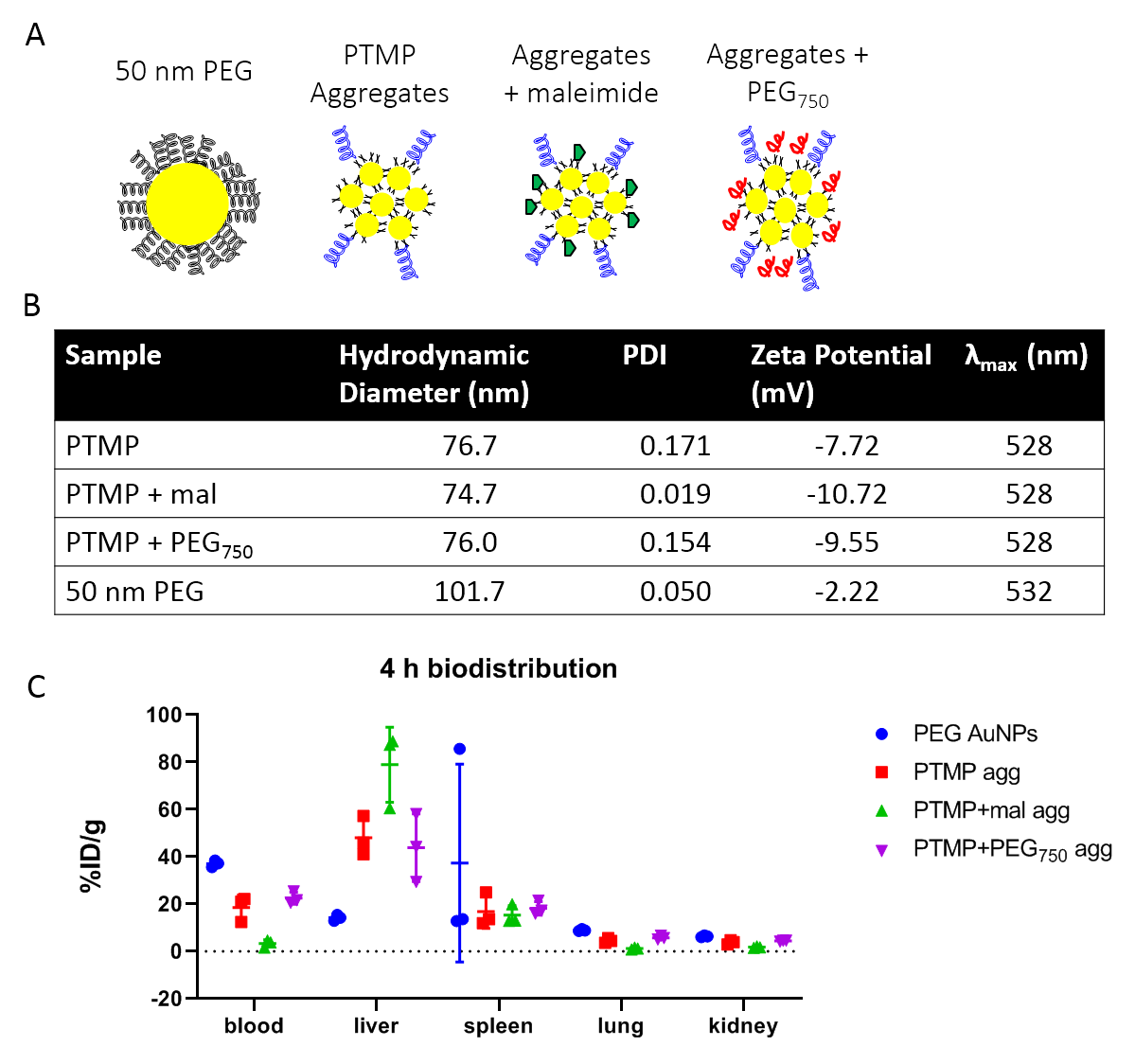
S8 Fig. Characterization and biodistribution of PTMP aggregates functionalized with 0.1x PEG_20k_-mal and backfilled with 1x maleimide or PEG_750_-mal, and AuNPs functionalized with 0.1x PEG_20k_-SH.** A) Hydrodynamic diameter and PDI were measured by DLS, surface charge was measured by zeta potential, and surface plasmon resonance peak (λ_max_) was measured by UV-vis absorption. B) Amount of gold measured by ICP-MS after 4 h in the blood, liver, spleen, lungs, and kidneys, reported as percent injected dose per gram of tissue. Error bars represent standard deviation, n=3.

**
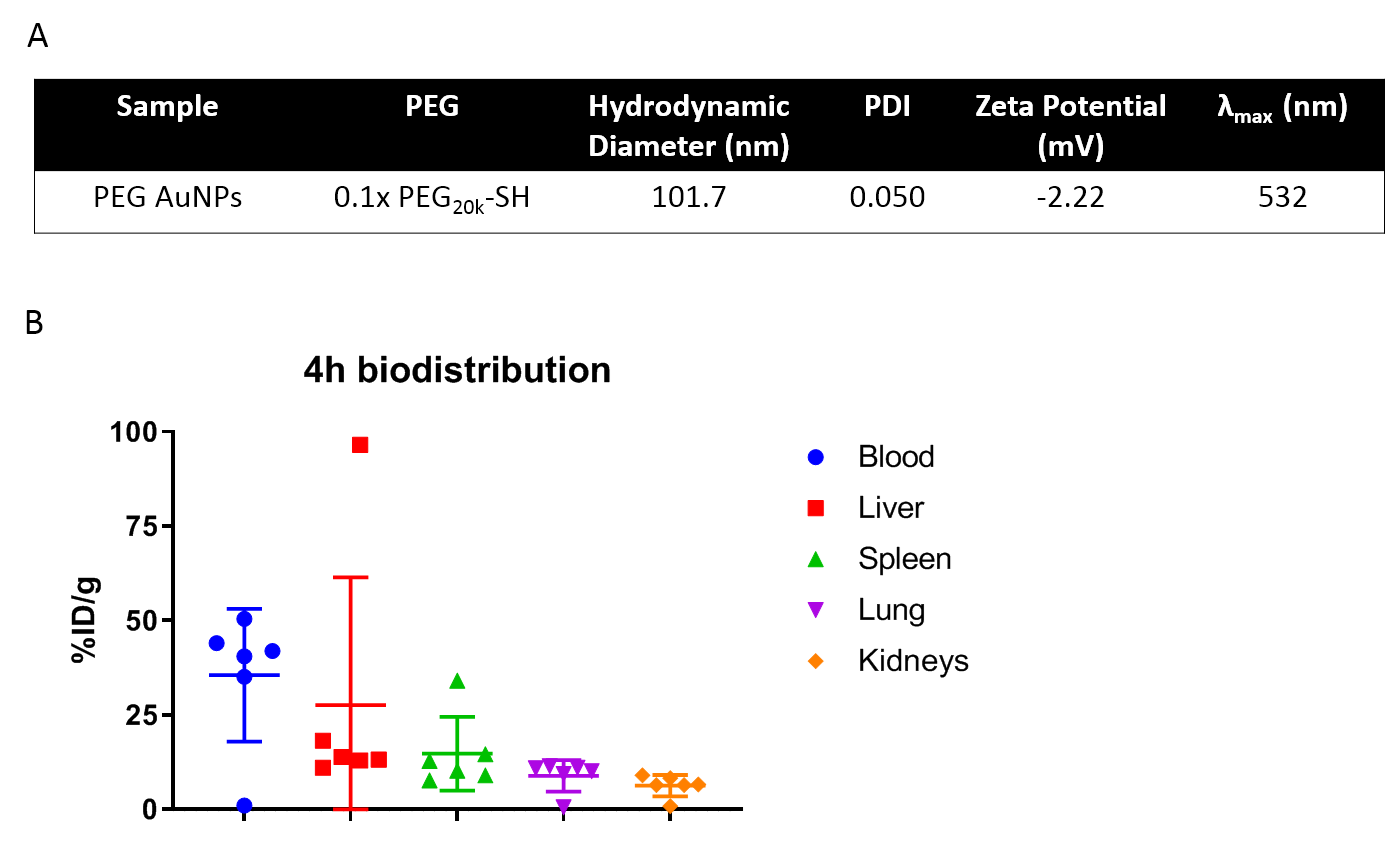
S9 Fig**. **Biodistribution of AuNPs functionalized with 0.1x PEG_20k_-SH in fasted mice.** Mice were fasted for 16 h before particle administration, and continued fasting for 4 h post-injection. Amount of gold in the blood, liver, spleen, lung, and kidneys were measured by ICP-MS at 4 h post-injection, reported as percent injected dose per gram of tissue. Error bars represent standard deviation, n=6.

| A) | 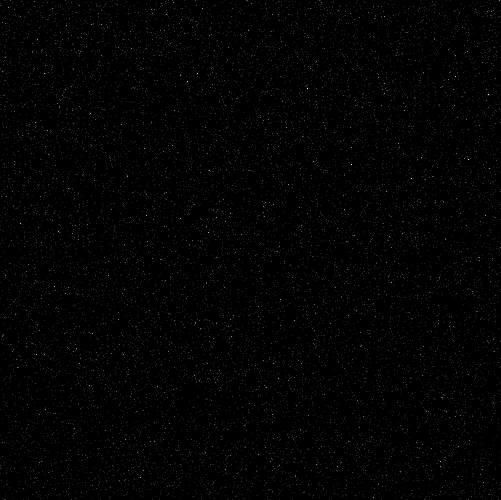 |
| --- | --- |
| B) | 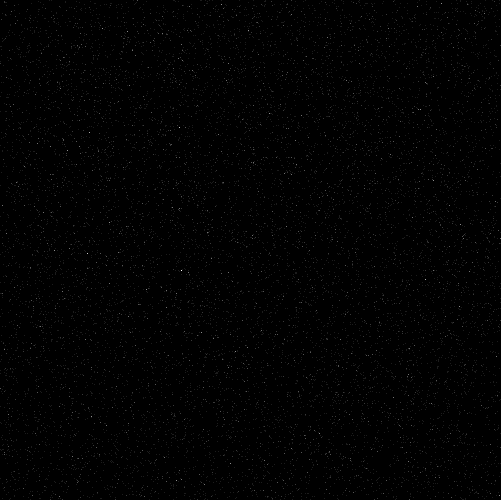 |

**S10 Fig**. **Imaging mass cytometry images of PBS-treated liver sections (500 µm x 500 µm) at the 4 h time point.** Gold distribution (white dots) in either a perivascular (A) or midzonal (B) region are shown.

1. Perrault SD, Walkey C, Jennings T, Fischer HC, Chan WCW. Mediating Tumor Targeting Efficiency of Nanoparticles Through Design. Nano Letters. 2009 2009/05/13;9(5):1909-15.

2. Maldiney T, Richard C, Seguin J, Wattier N, Bessodes M, Scherman D. Effect of core diameter, surface coating, and PEG chain length on the biodistribution of persistent luminescence nanoparticles in mice. ACS Nano. 2011 Feb 22;5(2):854-62. PubMed PMID: 21291197. Epub 2011/02/05. eng.

3. Sitaula S, Mackiewicz MR, Reed SM. Gold nanoparticles become stable to cyanide etch when coated with hybrid lipid bilayers. Chemical communications (Cambridge, England). 2008 Jul 14(26):3013-5. PubMed PMID: 18688332. Epub 2008/08/09. eng.

4. Van Haute D, Liu AT, Berlin JM. Coating Metal Nanoparticle Surfaces with Small Organic Molecules Can Reduce Nonspecific Cell Uptake. ACS Nano. 2018 2018/01/23;12(1):117-27.

5. Perry JL, Reuter KG, Kai MP, Herlihy KP, Jones SW, Luft JC, et al. PEGylated PRINT nanoparticles: the impact of PEG density on protein binding, macrophage association, biodistribution, and pharmacokinetics. Nano Lett. 2012 Oct 10;12(10):5304-10. PubMed PMID: 22920324. Pubmed Central PMCID: PMC4157665. Epub 2012/08/28. eng.

6. Mosqueira VCF, Legrand P, Morgat J-L, Vert M, Mysiakine E, Gref R, et al. Biodistribution of Long-Circulating PEG-Grafted Nanocapsules in Mice: Effects of PEG Chain Length and Density. Pharmaceutical Research. 2001 October 01;18(10):1411-9.

7. Liu Y, Hu Y, Huang L. Influence of Polyethylene Glycol Density and Surface Lipid on Pharmacokinetics and Biodistribution of Lipid-Calcium-Phosphate Nanoparticles. Biomaterials. 2014 01/02;35(9):3027-34. PubMed PMID: PMC3926205.

8. Xia X, Yang M, Wang Y, Zheng Y, Li Q, Chen J, et al. Quantifying the Coverage Density of Poly(ethylene glycol) Chains on the Surface of Gold Nanostructures. ACS Nano. 2012 2012/01/24;6(1):512-22.

9. Dai Q, Walkey C, Chan WCW. Polyethylene Glycol Backfilling Mitigates the Negative Impact of the Protein Corona on Nanoparticle Cell Targeting. Angewandte Chemie International Edition. 2014;53(20):5093-6.
